# Genetic abnormalities in biopsy-proven, adult-onset hemolytic uremic syndrome and C3 glomerulopathy

**DOI:** 10.1007/s00109-021-02102-1

**Published:** 2021-10-29

**Authors:** Ludwig Haydock, Alexandre P. Garneau, Laurence Tremblay, Hai-Yun Yen, Hanlin Gao, Raphaël Harrisson, Paul Isenring

**Affiliations:** 1 Nephrology Research Group, L’Hôtel-Dieu de Québec Research Center, Department of Medicine, Faculty of Medicine, Laval University, Quebec, QC G1R2J6 Canada; 2 Cardiometabolic Axis, School of Kinesiology and Physical Activity Sciences, Faculty of Medicine, University of Montréal, 900, rue Saint-Denis, Montreal, QC H2X 0A9 Canada; 3 Fulgent Genetics, Temple City, CA 91780 USA

**Keywords:** Atypical uremic syndrome, Thrombotic microangiopathy, C3 glomerulopathy, Alternative complement pathway, Genetic testing

## Abstract

**Abstract:**

Atypical hemolytic uremic syndrome (aHUS) and C3 glomerulopathy (C3G) have been linked to mutations in many of the proteins that are involved in alternative complement pathway activation. Age and etiology confounded, the prevalence of such mutations has been reported to be over 30 to 50% in these diseases. However, the cohorts studied included many children or individuals with a familial history of complement-related disorders and genetic tests were usually limited to exome sequencing of known causative or risk-associated genes. In this study, a retrospective adult cohort of 35 patients with biopsy-proven thrombotic microangiopathy (the largest in Canada) and 10 patients with C3 glomerulopathy was tested through an extended exome panel to identify causative defects in associated or candidate genes including those of the alternative and terminal complement pathways. A variant of unknown significance was also analyzed for pathogenicity through in vitro studies. To our surprise, the prevalence of known causative or risk-associated variants in either of these cohorts was found to be less than ~ 15% overall. However, the panel used and analyses carried out allowed to identify novel variants of potential clinical significance and a number of candidate genes. The prevalence of known genetic defects in adult-onset aHUS and C3G is thus probably much lower than 30 to 50%. Our results also point towards the importance of investigating diseases of the alternative complement pathway through extended exome panels and in vitro analyses.

**Key messages:**

- The alternative complement pathway plays a major role in the pathogenesis of hemolytic uremic syndrome and C3 glomerulopathy.
- Based on previous studies, both disorders have been commonly linked to variants in the various intermediates that sustain or regulate this pathway.
- The prevalence of such mutations in the adult-onset and sporadic forms of these diseases is probably much lower than expected based on larger series.
- The sporadic forms of complementopathies are likely to involve additional genes that are yet to be uncovered.

**Supplementary information:**

The online version contains supplementary material available at 10.1007/s00109-021-02102-1.

## Introduction

The alternative complement pathway plays a major role in the pathogenesis of atypical hemolytic uremic syndrome (aHUS) [1, 2], and it appears to do so whether the disorder is idiopathic or due to acquired conditions (such as those listed in Supplemental Table S1) [1, 3–8]. In the International Registry of Recurrent and Familial HUS [1], for instance, ~ 45% of the individuals who were affected by primary aHUS and ~ 30% who were affected by secondary aHUS were found to carry a pathogenic variant in a number of the proteins that sustain or regulate C3b-dependent C5b-9 deposition [1].

The pathological lesion of primary and secondary aHUS is known as thrombotic microangiopathy (TMA). It consists principally of tumefied endothelial cells and platelet-based fibrinous thrombi in the microcirculation [9]. The same lesion is seen in typical or Shiga toxin–mediated HUS (STEC-HUS) and in thrombotic thrombocytopenic purpura (TTP). Interestingly, the alternative complement pathway could also play an important pathogenic role in these other TMA-causing diseases [1, 6, 7, 10–15].

C3 glomerulopathy (C3G) is another disorder in which the alternative complement pathway is typically overactive [16]. However, the pathological lesion in this disorder is the presence of C3-based, IgG-free deposits in the glomerulus along with mesangial or endothelial proliferation in most cases [8, 17–20]. C3G also comes as two histological variants, i.e., C3 glomerulonephritis and dense deposit disease, and can also occur in the setting of underlying conditions such as monoclonal gammopathies and infections [21].

As it stands, the true prevalence of mutations in adult-onset HUS and C3G is uncertain. It could be underestimated as genetic tests are typically limited to exome sequencing of known causative or risk-associated genes and because variants of unknown significance (VUS) are seldom tested for pathogenicity. The prevalence of mutations in adult-onset HUS and C3G could be overestimated as well if, for instance, it was extrapolated from a population that included a high percentage of children or patients with a familial history of complement-related disorders.

The Centre hospitalier universitaire de Québec acts as a specialized adult Nephrology Service for a population of over 1.5 million. Through this role, it has managed more than 100 cases of HUS and C3G in the last 25 years and has investigated many of these cases through serum ADAMTS13 measurements, kidney biopsies, and extended exome panels. It now counts the largest Canadian cohort of adult patients who were diagnosed with and genotyped for biopsy-proven TMA.

In the current study, a general description of these two cohorts is provided through clinico-biological parameters while emphasizing on the genetic abnormalities identified. As the participants investigated were over 18 of age and as they were all included in the study based on robust selection criteria, a number of findings came as quite unexpected.

## Methods

### Cohort description

The cohort at hand included 45 patients who were all investigated and treated at the L’Hôtel-Dieu de Québec Hospital (of the CHU de Québec). Thirty-five such patients were diagnosed with HUS (*n* = 35) between 1990 and 2019 (30 after 2010), and ten were diagnosed with C3G (*n* = 10) between 2007 and 2019 (9 after 2012). The data presented in this study were obtained in the context of optimal care regimens and not of pre-existing or ongoing research protocols. HUS or C3G were arbitrarily defined as “secondary” if they occurred in the setting of a condition that has been linked to these disorders.

Inclusion criteria were as follows: (1) age ≥ 18, (2) evidence of renal TMA or C3G based on histological reports, and (3) availability of genetic tests. Exclusion criteria were as follows: (1) serum ADAMTS13 activity < 10% and (2) evidence for disseminated intravascular coagulation. TMA was diagnosed more specifically in the presence of (1) endothelial tumefaction associated or not with intraluminal fibrin thrombi or (2) unexplained chronic microvascular ischemic injury such as glomerular tuft retraction, double-contoured basement membranes and mesangiolysis [22]. As for C3G, it was diagnosed in the presence of C3-based IgG-free deposits in the glomerulus [17].

Note that a reassessment of the histopathological lesions described in the clinical charts was not carried out for the current study as the initial renal biopsies or slides were not available for all patients. It is however unlikely that such a reassessment would have been of great value. In particular, the histological reports were unequivocal and the usefulness of pathological data to discriminate among the various causes of HUS or C3G is speculative rather than evidence-based [23–25]. Additionally, C3G was diagnosed after 2007 in all patients, i.e., during a period of time when the diagnostic criteria of C3G were already established.

### Data collected

#### General characteristics

Demographic parameters, clinical manifestations, and laboratory results (baseline as well as etiology-oriented) were obtained through systematic chart reviews. In some cases, additional laboratory results were obtained through the provincial database Dossier Santé Québec (DSQ). All of the data reported under this section had been collected while HUS or C3G was active and while none of the patients had received plasma exchange therapy or anti-C5 antibodies.

#### In-depth biochemical characterization of the alternative complement pathway

Many of the patients recruited had also been subjected to one or several of the following assays: serum concentration of factor H, B, I, C5b-9, and anti-CFH antibodies, serum activity of plasminogen and C3NEF, as well as urinary concentration of C5B-9. Once again, all samples had been collected while none of the patients had received plasma exchange therapy or anti-C5 antibodies for their condition.

Assays used for certain of the measurements were as follows:

1. Anti-CFH antibodies were measured by ELISA (CHU Sainte-Justine, Montreal, Canada).
2. C3NEF activity was measured through a red blood cell (RBC) hemolysis test in which the patient’s serum is incubated with sensitized sheep RBC in the presence of EGTA or EDTA and in which data are expressed as optical density (proportional to RBC count) with EGTA/optical density with EDTA (CHU de Quebec, Quebec, Canada).
3. CFB and CFH concentrations were measured by radial immunodiffusion (CHU de Quebec, Quebec, Canada).
4. CFI concentration was measured by competitive ELISA (kit from Abcam, Cambridge, UK).
5. Urinary and serum C5b-9 concentration were measured by ELISA (kit from Quidel, San Diego, CA).

#### Genetic characterization of the alternative complement pathway

##### Data collection

**Fig. 1:**
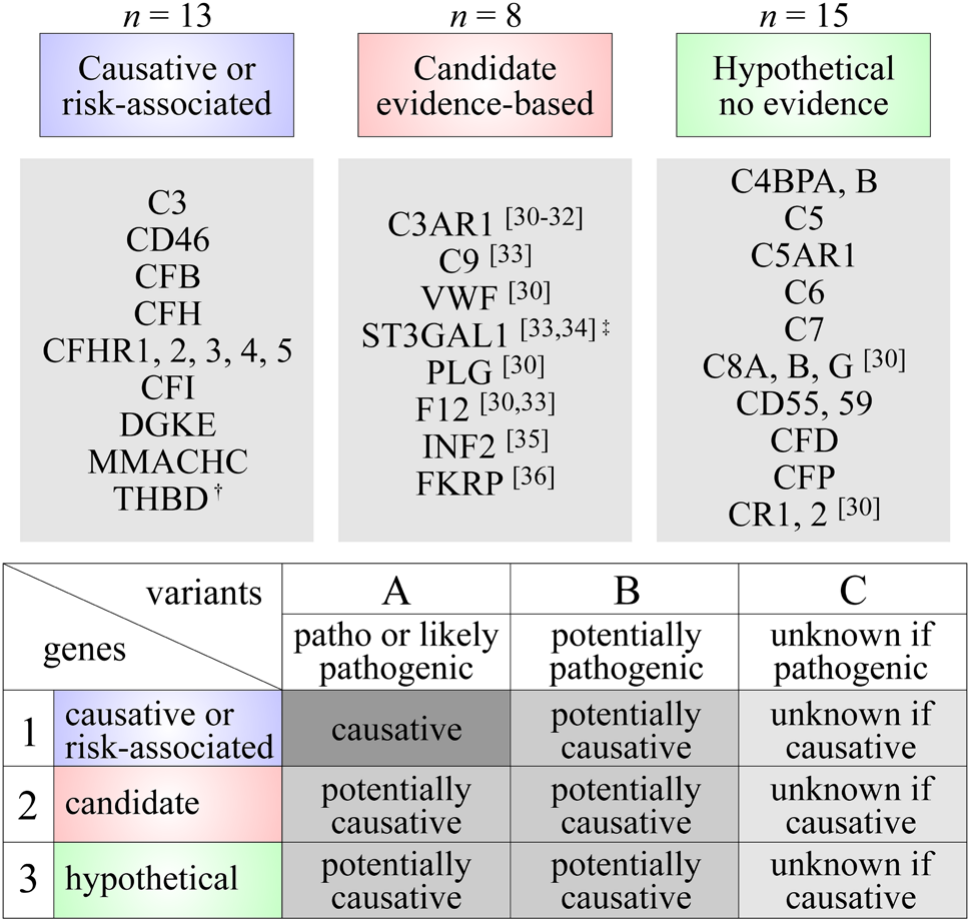
Genetic analyses. (**A**) Genetic testing. All patients were subjected to an exome panel of 36 genes. These genes have been linked to HUS and/or C3G based on various levels of evidence or could be linked to such diseases based on hypothetical grounds. They were ascribed to three groups as follows: 1, causative or risk-associated (*n* = 13); 2, candidate (*n* = 8); and 3, hypothetical (*n* = 15). Note that the CFHR3-1 deletion is a common polymorphism (allele frequency up to 18% in certain populations) in the general population but considered to be a risk factor for diseases of the alternate complement system [50, 51]. Also note that the sequencing approach used allowed for the detection of CFH/CFHR hybrid genes but not of aHUS-associated haplotypes in CFH and CD46 [52–57]. (**B**) Categorization of variants. Variants were also ascribed to three groups (**A**, **B**, or **C**) as follows: (**A**) pathogenic or likely pathogenic if known to disrupt protein function, (**B**) potentially pathogenic if suspected to disrupt protein function based on the criteria specified in the “Methods” section, and (**C**) unknown if pathogenic. The variants were further categorized based on their likelihood to cause the observed phenotype, i.e., as causative, potentially causative and unknown if causative depending on pathogenicity and genes in which they were identified. Signs: dagger, the evidence linking THBD to diseases of the alternative complement system is no longer considered strong based on recent studies [58]; double dagger, recessive mutations leading to loss of ST3GAL1 activity have been identified in three siblings with aHUS [33, 34]

Genetic testing was performed in all patients on genomic DNA extracted from peripheral blood. For some patients, the DNA samples used were not collected at presentation or they were used in a second round of analyses to sequence additional genes. In the end, the gene panel was still the same for all of the 45 subjects included in the study (see below and Fig. 1).

##### Method used to identify the variants

Exons and exon-flanking intronic segments (~ 200 base pairs on each side) were sequenced by the company Fulgent (Temple City, CA) through a next generation sequencing (NGS) approach at a minimum coverage of 20 × . A deletion/duplication analysis was also carried out by NGS at the same coverage and at a resolution of ~ 200 base pairs or ~ 2 exons. This approach has proven successful to manage genetic analysis in the presence of homologous sequences [26] and provides sensitivities/specificities for detection of copy number variations that are comparable to other approaches such as aCGH and qPCR [27–29].

##### Complementome

Genes for which exons and exon-flanking intronic sequences were obtained are listed in Fig. 1. In all patients, they included those that have been linked to HUS or C3G as causative or risk-associated genes (group 1; *n* = 13) and as candidate genes based on emerging evidence (group 2; *n* = 8)—see refs. [30–36] in this regard. They also included hypothetical genes that were added to the panel because of their implication in the alternative complement pathway (group 3; *n* = 15).

##### Interpretation of genetic abnormalities

As shown in Fig. 1, three categories of variants were considered, i.e., pathogenic or likely pathogenic if known to disrupt protein function (type A), potentially pathogenic (type B), and unknown if pathogenic (type C). Group B included more specifically variants that occurred at a maximal allele frequency of less than 0.02% in the general population and that also met at least one other criterion, i.e., (i) were observed in other patients with HUS or C3G, (ii) are predicted to disrupt pre-mRNA splicing, (iii) caused a residue to be replaced by a highly divergent one (Grantham distance > 100), or (iv) caused a residue or several residues to be deleted. Variants were further categorized as “causative” based on current guidelines (group 1) [37] or “potentially causative” (group 2 and 3). Type B variants were thus all considered “potentially causative.”

##### In vitro analysis

Based on multiple splice site finders, one of the variants identified in C3 was predicted to result in the creation of an aberrant 5′ donor splice site 205 bps downstream of exon 29. This possibility was tested through RT-PCR using C3-specific primers (5′: GCTAAAAGACTTTGACTTTGTG; 3′: GCAGTTGGAGGGACACATCAAG) and leukocyte-derived mRNA (known to include C3 transcripts^1^) from both the patient and two healthy but unrelated volunteers. The 5′ primer was designed to anneal with a segment of exon 29 and the 3′ primer with a segment of exon 30. The templates used were freed of genomic DNA before amplification, and the cDNAs obtained were characterized by gel electrophoresis and automated sequencing.

### Statistics

Data are presented as means ± SD or medians (IQR), percentages, or fold differences between the upper or lower limit of a reference interval. Relative risk factors for certain outcomes were calculated as hazard ratios and statistical relationships between sets of variables through Pearson product-moment correlation coefficients.

### Ethical issues

All protocols and methods were approved by the Comité d’éthique de la recherche (CER) du CHU de Québec in accordance to relevant guidelines and regulations. Informed consent was also obtained from all participants to be included in the study and subjected to DNA testing.

## Results

### Cohort at hand and summary of study design

The cohort under study included 35 patients with biopsy-proven TMA and 10 patients with C3G. Those with decreased ADAMTS13 activity were excluded from the populations examined. The initial investigation had been carried out after 1990 for the cases of HUS and after 2007 for the cases of C3G. Otherwise, the histological data at hand did not point towards specific etiologies as mentioned [23–25].

All patients were subjected to routine laboratory assays and an extended complement exome panel. The genes tested are listed in Fig. 1, and the abnormalities identified were categorized as described in “Methods” and illustrated again through Fig. 1 [37]. Many patients were also subjected to specialized assays aimed at characterizing the function of their alternative complement pathway in greater detail, and one patient was tested for pre-mRNA splicing defects through RT-PCR.

### Cohort analysis of patients with HUS

#### Baseline characteristics of individual participants

A relevant demographical and clinico-biological portrait of each patient who was recruited in this study is depicted through Table 1. The laboratory data shown were those of the inaugural investigation as well as the highest or lowest ones observed (LDH or haptoglobin, platelet count, C3, and eGFR, respectively). Many of the determinations are presented as fold changes to control for differences in reference intervals among the assays used during the study period.

**Table 1:** Selected demographical and clinical data in cohort with HUS. Cases are sorted by age at presentation. Some of the measurements are shown as fold differences between upper limit of normal range (LDH) or lower limit of normal range (HG, PLT, C3)

Case (#)	Age (years)	Gender (F/M)	LDH adm (fold)	LDH max (fold)	HG adm (fold)	HG min (fold)	PLT adm (fold)	PLT min (fold)	C3 adm (fold)	C3 min (fold)	eGFR min (mL/min)	Associated condition	*n* of variants
1	18	F	3.8	3.8	0.24	0.24	0.61	0.38	N	no AN	15	None	0
2	22	F	8.7	8.7	0.40	0.40	0.30	0.30	−	−	9	None	1^a^
3	24	F	1.5	1.8	N	0.88	N	0.64	−	0.71	27	Pregnancy	0
4	25	F	5.0	5.0	0.40	0.40	0.29	0.19	N	no AN	10	None	1^b^
5	27	F	5.9	5.9	0.29	0.29	0.28	0.28	N	no AN	12	Pregnancy	1^a^
6	27	F	5.0	5.0	0.16	0.16	0.37	0.37	N	no AN	8	Malignant HT^d^	1^a^
7	31	F	1.3	1.3	0.24	0.24	N	0.83	0.90	0.90	9	None	0
8	31	F	3.3	3.3	0.24	0.24	0.14	0.14	N	no AN	28	Pregnancy	2
9	35	F	5.1	5.1	0.29	0.29	0.40	0.40	N	no AN	28	IVIG for DM	3^b^
10	36	M	2.5	2.5	0.16	0.16	0.47	0.23	−	0.94	57	Colitis/CNI	2^b^
11	38	M	1.7	1.7	0.24	0.24	N	no AN	N	no AN	32	Severe HT^d^	0
12	39	F	7.8	7.8	0.29	0.29	0.39	0.39	N	no AN	3	Malignant HT^d^	1
13	41	M	2.0	2.0	N	no AN	N	no AN	N	no AN	9	Severe HT	2
14	44	F	N	no AN	0.92	0.36	N	no AN	N	no AN	32	Severe HT	1
15	47	F	1.4	1.4	N	no AN	0.65	0.65	−	0.99	15	Breast cancer^c^	0
16	48	F	6.0	6.0	0.21	0.21	0.15	0.09	−	0.88	13	None	0
17	48	M	2.4	2.4	0.16	0.16	0.53	0.53	−	0.59	6	Severe HT^d^	0
18	49	F	1.3	1.3	0.89	0.89	0.73	0.61	N	no AN	15	APLS	1^a^
19	50	M	2.0	2.1	0.11	0.02	0.64	0.53	N	no AN	31	IgA nephropathy	2^a^
20	50	F	7.9	7.9	0.16	0.16	0.21	0.21	N	no AN	53	Interferon β-1α	2^a^
21	52	F	7.5	7.5	0.02	0.02	0.33	0.33	−	0.77	7	None	2^a^
22	54	F	4.4	4.4	0.16	0.16	0.15	0.07	0.82	0.7	18	CNI	0
23	55	F	0.7	1.1	N	0.92	N	0.98	N	no AN	28	None	0
24	55	F	5.5	5.5	0.16	0.16	0.89	0.60	0.69	0.69	11	Interferon beta-1α	0
25	56	M	1.1	1.1	N	0.16	N	0.83	N	no AN	28	Severe HT^d^	2^a^
26	57	M	5.6	5.6	0.16	0.16	0.33	0.24	−	no AN	11	Prostate cancer	2^b^
27	58	F	1.1	1.4	−	no AN	N	no AN	N	no AN	18	None	0
28	58	M	6.5	6.5	−	0.73	0.82	0.71	−	−	6	Severe HT^d^	1^b^
29	61	F	2.9	2.9	0.82	0.82	0.33	0.28	−	0.97	10	None	1^a^
30	63	M	1.4	1.4	0.1	0.10	0.61	0.23	−	0.92	18	CNI	2
31	67	M	12.8	12.8	0.16	0.16	0.24	0.24	−	−	15	STX	1^b^
32	70	M	N	no AN	N	0.81	N	0.84	N	no AN	11	BM transplant	0
33	80	M	1.3	1.7	N	0.16	0.36	0.10	0.16	0.16	10	Pneumonia	0
34	81	F	12.6	12.6	0.16	0.16	0.12	0.12	N	no AN	11	None	0
35	83	M	1.5	1.5	N	no AN	N	no AN	N	no AN	20	None	4^a^

# code assigned to case, − data unavailable

*adm* on admission, *APLS* anti-phospholipid syndrome, *BM* bone marrow, *CNI* calcineurin inhibitors, *DM* dermatomyositis, *eGFR* estimated glomerular filtration rate based the CKD-EPI, *F* female, *HG* haptoglobin, *HUS* hemolytic uremic syndrome, *IVIG* intravenous immunoglobulins, *LD* lactate dehydrogenase, *M* male, *max* maximal, *min* minimal, *N* normal, *no AN* no abnormal values based on > 2 measurements, *PLT* platelets

^a^Presence of at least one potentially causative variant; ^b^Presence of at least one causative variant; ^c^Patient also had increased anti-nuclear antibody titre (1:2560) of unknown etiology; ^d^Funduscopic examination available in patients with severe or malignant HT

The etiological investigation carried out for each of the participants is detailed in Supplemental Table S2. Importantly, all patients who presented with diarrhea had their stools examined for the presence of Shiga toxins STX1 and STX2, patients who were not tested for both serum ANA and anti-ENA antibody titers had no clinical evidence or histological signs (on their renal biopsies) of autoimmune disorders, and six of the patients who were diagnosed with severe or malignant hypertension were subjected to funduscopic examinations.

#### Baseline characteristics of the cohort

Data are summarized in Table 2. As can be seen, the typical presentation was that of a 48-year-old woman with secondary HUS. It was also characterized by high LDH serum levels in 94% of patients, C3 consumption in 38%, and dialysis-requiring renal failure in 77%. Note that 100% of patients were affected by a non-familial form of HUS, 31% were considered to have primary aHUS, 57% were treated with an anti-C5 antibody, and 6% were deceased due to HUS-related complications. Also note that among the patients who were diagnosed with primary aHUS, half presented with a non-specific infectious illness within the month that preceded admission.

**Table 2:** Baseline characteristics of cohort with HUS. Unless indicated otherwise, values shown are based on the total number of patients for whom data were available

Characteristics	*n* = 35
Age at diagnosis (y.o.)	
Mean ± SD	48 ± 17
Median (IQR)	49.0 (18–83)
Female (%)	62.9
Etiology	
Idiopathic (%)	31.4^a^
Hypertension (%)	22.9
Family history (*n*)	0.0
LDH max ↑ (%)	94.3
HG min ↓ (%)	88.6
Platelets min ↓ (%)	85.6
C3 min ↓ (%)	37.5^b^
eGFR min: n (%)	
> 90	0 (0)
60–89	0 (0)
45–59	2 (5.7)
30–44	3 (8.6)
15–29	13 (37.1)
< 15	17 (48.6)
< 29	30 (85.7)
Dialysis: *n* (%)	27 (71.4)
Transplantation: *n* (%)	11 (31.4)
Anti-C5 Ab: *n* (%)	20 (57.1)
Death: *n* (%)	2 (5.7)

*Ab* antibody, *eGFR* estimated glomerular filtration rate based on the CKD-EPI equation, *HG* haptoglobin, *LD* lactate dehydrogenase, *max* maximal, *min* minimal, *n* number, *SD* standard deviation

^a^Half of patients presented with non-specific symptoms; ^b^Unavailable in three patients

**Fig. 2:**
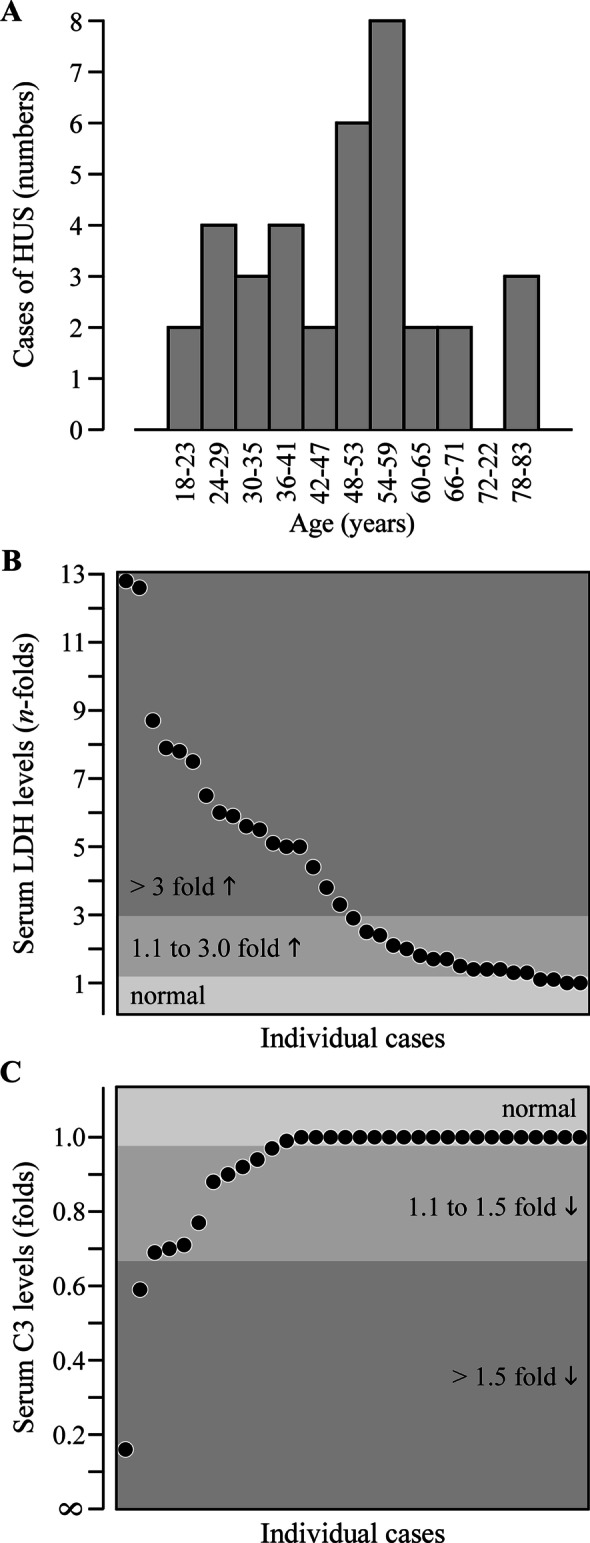
Distribution analysis of selected clinical parameters in the cohort affected by HUS. **A** Age at presentation. **B** Maximal LDH serum levels. **C** Minimal serum C3 levels

In Fig. 2, a distributional analysis is shown for three parameters of interest. It is seen that the number of patients with HUS is evenly dispersed among the age groups (panel A) and that LDH and C3 serum levels vary widely among subjects (panels B and C). It should be noted, however, that when LDH serum levels are abnormal, they differ from baseline by less than 3.0 folds in as much as 43% of patients.

#### In-depth biochemical analysis of the alternative complement pathway

Results are shown in Table 3. One can observe that serum and urinary C5b-9 are elevated in many patients (but not in all of them) and that all other measurements are abnormal in less than 34%. Somewhat surprisingly, two out of the four patients who were found to exhibit a mild decrease in CHF serum concentration carried a potentially causative variant in the encoding gene (see Table 3 footnote).

**Table 3:** In-depth investigation of cohort with HUS. Data shown are from all individuals who were included in the study

	*n* tested % of total	*n* abnormal % of tested	Mean *N* value
Factor H	28	4^a^	597 ± 109
	80	14.4	441–761 mg/L
Factor B	15	5^b^	373 ± 111
	42.9	33.3	173–453 mg/L
Factor I	8	0.	1.52 ± 0.42
	22.9	0.0	0.6–1.4 U/mL
C3NEF	17	0	7.2 ± 6.1
	48.6	0.0	0.0–19.0%
PLG	14	2	1.10 ± 0.19
	40	14.3	0.88–1.37 U/mL
C5b-9	12	11	901 ± 641
	34.3	91.7	< 300 ng/mL
Urinary C5b-9	4	3	159 ± 226
	11.4	75.0	< 15.0 ng/mL
Anti-CFH Ab	20	0	1.1 ± 1.5
	57.1	0.0	< 2.0 dilutions

*Ab* antibody, *C3NEF* C3 nephritic factor, *N* normal, *n* number, *PLG* plasminogen

^a^The decrease in CFH serum concentration was mild (400 to 435 mg/L) and associated with potentially causative CFH variants in only two of the patients; ^b^Abnormal value was defined as above the upper limit of normal range

#### Genetic abnormalities

As expected, and as shown through Supplementary Table S3, many variants were identified in the retrospective cohort. Based on various parameters and their characteristics, they were considered benign or likely benign in 37% of patients and they were considered of unknown significance, risk-associated, likely pathogenic or pathogenic in 63%. Among the latter group, and as depicted through Table 1, 31% of patients were also found to carry at least two variants in different alleles. Of note, the term “pathogenic” refers to a change in protein function (whether it be a decrease or an increase) and not to necessarily to a causality link between variant and disease.

**Fig. 3:**
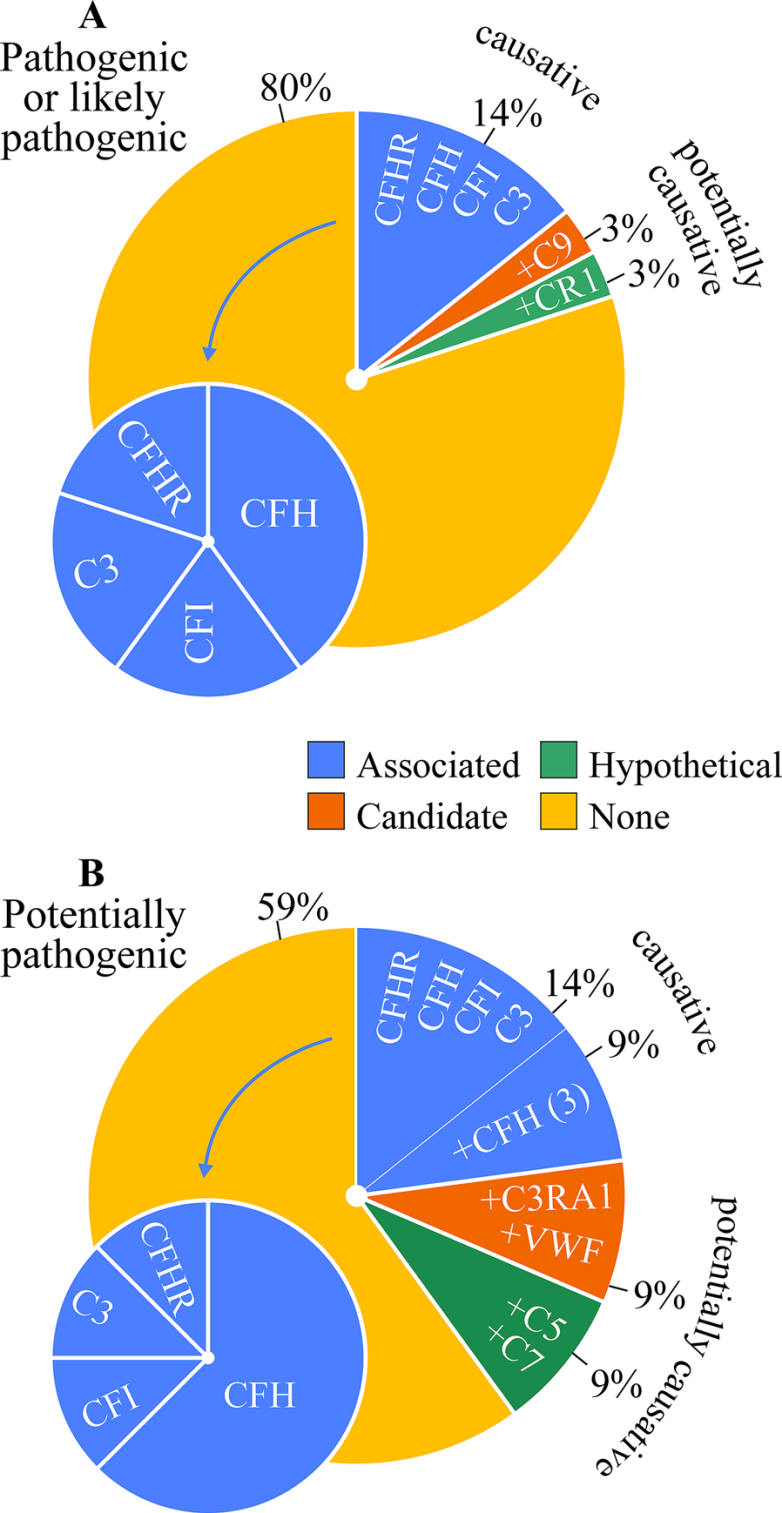
Distribution analysis of genetic variants identified in cohort with HUS. (A) Frequency of pathogenic, likely pathogenic or risk variants in genes tested. (B) Similar to (A) except for adding potentially pathogenic variants

The relative frequencies of the causative or potentially causative variants identified are illustrated through Fig. 3. As can be observed, only 14% of patients are found to carry pathogenic, likely pathogenic or risk variants in causative genes. This percentage increases to 20% when potentially causative variants in candidate genes (C9) or hypothetical genes (CR1) are included and to 41% when potentially pathogenic variants in causative genes (3 CFH; 1 CFB), candidate genes (1 C3AR1; 1 VWF), and hypothetical genes (1 C5; 1 C7) are included as well. The nature and characteristics of the variants identified are presented more specifically in Table 4.

**Table 4:**
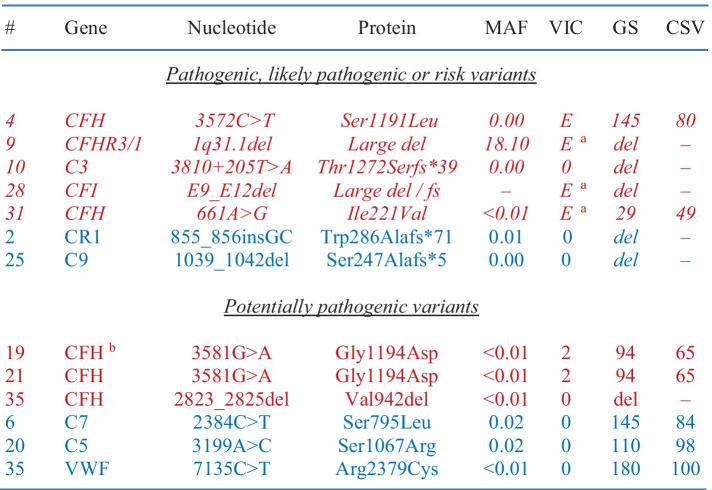
Variants of interest in cohort with HUS. Five of the variants identified (shown in italic characters) are known to be associated with aHUS and seven of the variants identified (shown in non-italic characters) were considered potentially causative (shown in red characters for associated genes and blue characters for candidate or hypothetical genes). In the Table, parameters used to analyze the significance of each variant are also provided in the last four columns. Note that the intronic variant in C3 is predicted to result in the creation of an aberrant 5' donor splice site based on multiple splice site finders. Also note that case #19 and #21 are unrelated

#code assigned to case; Font and color code: in italic, known to be associated with aHUS; not in italic, potentially causative; red, aHUS-associated genes; blue, candidate or hypothetical genes for HUS

*CSV* conservation (% of mammalian species among > 50 different ones in which native residue in conserved), *del* deletion, *E* established risk or disease-causing variant, *fs* frameshift, *GS* Grantham score, *MAF* minor allele frequency, *VIC* variant in cases (as previously reported)

^a^Increased risk for aHUS (> fivefold with the 1q31.1 deletion); ^b^Both alleles were affected by the same variant

Figure 3 is also used to show the frequencies of the uncovered causative or risk associated genes compared to one another (left blue pies on the bottom). It is seen that among these genes, the most commonly affected one is CFH whether or not the potentially pathogenic variants are included in the analysis. The prevalence of pathogenic variants in causative genes was not very different among the various etiological subgroups either, that is, 9% in primary aHUS vs. 17% in non-primary HUS and 13% in hypertension-related secondary aHUS vs. 15% in non-hypertension-related HUS (results not shown).

#### Molecular analysis of a C3 variant

**Fig. 4:**
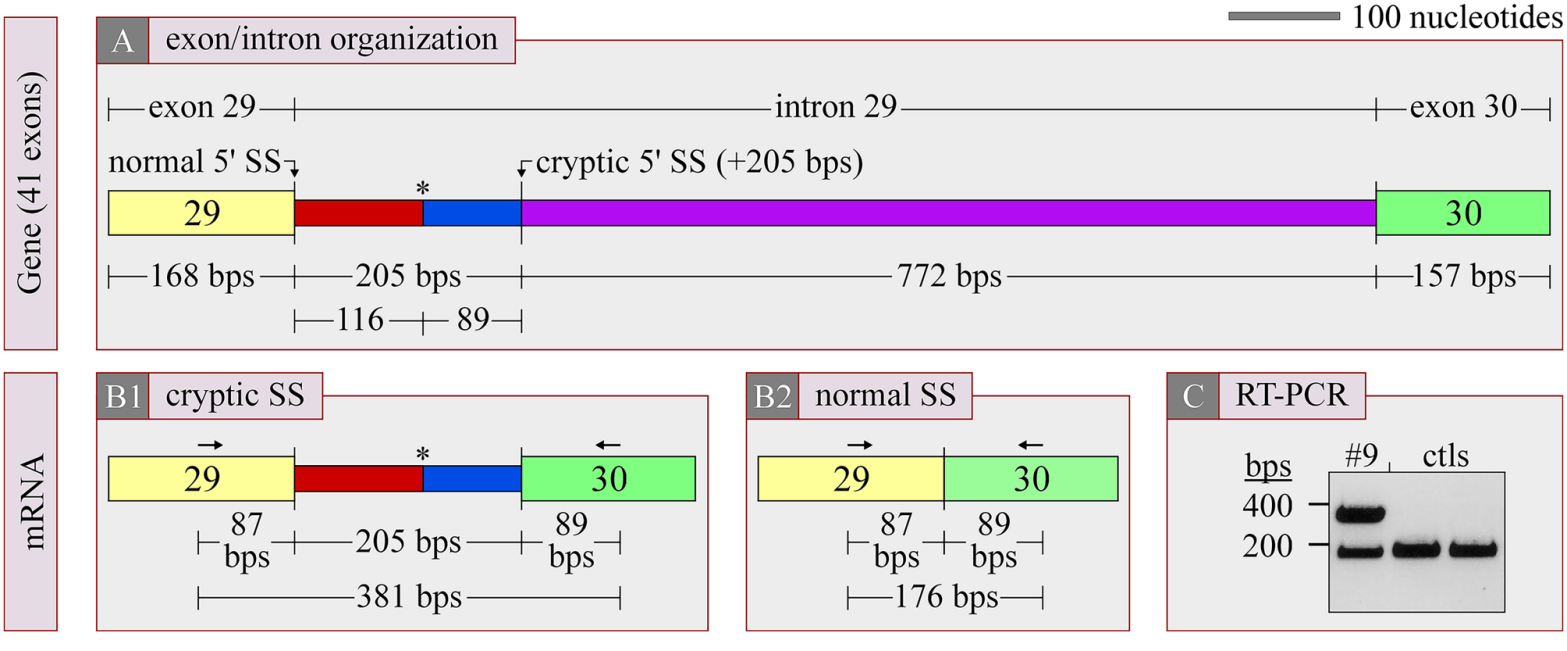
RT-PCR studies. Analysis of a VUS in C3. (A) Location of VUS in intron 29. (B) Predicted mRNA if VUS leads to aberrant splicing by creating a 5′ cryptic splice site (B1) and predicted mRNA if VUS does not do so (B2). Aberrant splicing should also translate into the addition of 38 neo-residues (ASGPTAPRHMHPCLLRLPTGLLEKTLRPSEAVLHSHEPV) at the end of the truncated gene product). (C) Ethidium bromide-stained agarose gel. Arrows: rightwards arrow, sense oligonucleotide; leftwards arrow, antisense oligonucleotide; asterisk, stop codon; bps, base pairs

As mentioned earlier, the potential of a monoallelic intronic variant to create an aberrant 5′ donor splice site in C3 was tested further through non quantitative RT-PCR using white blood cell mRNA as the template and exon-specific oligonucleotides. As illustrated through Fig. 4 (A and B), an intron-containing PCR product of 381 bps will be generated through the aberrant splice site and an intronless PCR product of 176 bps if the variant is inactive. Note that if the variant leads instead to the creation of an aberrant 3′ acceptor splice site, exon 30 will be excluded from the final mRNA and the 3′ oligonucleotide will thus have no template to anneal with.

In Fig. 4 (C), it is seen that the monoallelic variant appears to generate an aberrant 5′ donor splice site given that a PCR product of ~ 400 bps is observed for the patient only. The presence of a retained intronic sequence was ultimately confirmed through automated sequencing of the larger DNA fragment. As such, and because the added sequence disrupts the open reading fame, all or part of the affected allele codes for a protein that is devoid of the last 12 exons out of 41, i.e., for a type of C3 mutant that has been linked to aHUS as a dominantly inherited defect [38].

### Cohort analysis of patients with C3G

#### Baseline characteristics of individual participants

A relevant demographical and clinico-biological portrait of each patient is summarized in Table 5. The laboratory data shown were those of the investigation carried out initially and the lowest ones observed (C3 and eGFR). Many of the determinations are presented once again as fold changes to control for differences in reference ranges among the assays used during the entire study period. Otherwise, the etiological investigation carried out for each of the participants is detailed in Supplementary Table S4

**Table 5:** Selected demographical and clinical data in cohort with C3G. Cases are sorted by age at presentation. Some of the measurements are shown as fold differences between lower limit of normal range (PLT, C3, C4)

Case (#)	Age (years)	Gender (F/M)	Proteinuria (g/mM)	PLT (fold)	C3 max (fold)	C3 min (fold)	C4 min (fold)	eGFR min (mL/min)	Associated condition	*n* of variants
1	18	M	1.23	N	N	0.22	N	107	None	2^b^
2	19	F	1.17	N	N	0.22	N	< 10	None	1^a^
3	22	M	0.33	N	N	0.14	N	< 10	None	1
4	44	M	0.35	N	N	0.84	N	98	None	2
5	46	F	0.45	N	N	no AN	N	62	None	2^b^
6	56	M	0.16	N	−	−	−	< 10	Gammopathy	1^a^
7	58	M	0.33	N	N	no AN	N	16	HIV	0
8	61	M	0.32	N	N	0.78	N	< 10	Gammopathy	0
9	68	F	0.58	N	N	no AN	N	23	Gammopathy	2
10	74	F	0.09	N	N	0.88	N	18	Gammopathy	0

#code assigned to case, − data unavailable

*eGFR* estimated glomerular filtration rate based the CKD-EPI, *F* female, *M* male, *max* maximal, *min* minimal, *N* normal, *no AN* no abnormal values based on > 2 measurements, *PLT* platelets

^a^Presence of at least one potentially causative variant; ^b^Presence of at least one causative variant

#### Baseline characteristics of the cohort

Data are summarized in Table 6. Here, and in keeping with previous observations, the typical presentation was that of a 51-year-old male with monoclonal gammopathy [21]. It was also characterized by nephrotic range proteinuria in 80% of patients, C3 consumption in 67%, and dialysis-requiring renal failure in 40%. In addition, 40% of patients (*n* = 4) were treated with an anti-C5 antibody. In two of them, a spectacular response was observed and, in the other two, the response was null (not shown).

**Table 6:** Baseline characteristics of cohort with C3G. Unless indicated otherwise, values shown are based on the total number of patients for whom data were available

Characteristics	*n* = 10
Age at diagnosis (y.o.)	
Mean ± SD	47.0 ± 20.1
Median (IQR)	51.0 (18–74)
Female (%)	40.0
Etiology	
Idiopathic (%)	50.0
Gammopathy (%)	40.0
Family history (*n*)	0.0
UP > 0.25 g/mM CR (%)	80
C3 min ↓ (%)	66.7^a^
eGFR min: *n* (%)	
> 90	2 (20.0)
60–89	1 (10.0)
45–59	0 (0.0)
30–44	0 (0.0)
15–29	3 (33.3)
< 15	4 (40.0)
< 29	7 (70.0)
Dialysis: *n* (%)	4 (40.0)
Transplantation: *n* (%)	1 (10.0)
Anti-C5 Ab: *n* (%)	4 (40.0)
Death: *n* (%)	0 (0.0)

*Ab* antibody, *CR* creatinine, *eGFR* estimated glomerular filtration rate based on the CKD-EPI equation, *max* maximal, *min* minimal, *n* number, *SD* standard deviation

^a^Unavailable in one patient

#### In-depth biochemical analysis of the alternative complement pathway

Results are shown in Table 7. One can observe that serum C5b-9 are elevated in all of the patients tested and that anti-CFH antibodies are detected in two of them but that all other measurements are abnormal in less than 34%. Of note, the two patients who were found to exhibit a decrease in CFH serum concentration carried no variants in the encoding gene and were not the ones who tested positive for the presence of ant-CFH antibodies in their serum (see Table 7 footnote).

**Table 7:** In-depth investigation of cohort with C3G. Data shown are from all individuals who were included in the study

	*n* tested % of total	*n* abnormal % of tested	Mean *N* value
Factor H	9	2^a^	532 ± 129
	90.0	22.2	441–761 mg/L
Factor B	5	0^b^	329 ± 91
	50.0	0.0	173–453 mg/L
Factor I	1	0	0.99 (n = 1)
	10.0	0.0	0.6–1.4 U/mL
C3NEF	9	1	14.1 ± 25.5
	90.0	11.1	0.0–19.0%
PLG	2	0	1.05 ± 0.05
	20.0	0	0.88–1.37 U/mL
C5b-9	5	5	1019 ± 749
	50.0	100.0	< 300 ng/mL
Anti-CFH Ab	5	2^c^	3.2 ± 2.9
	50.0	40.0	< 2.0 dilutions

*Ab* antibody, *C3NEF* C3 nephritic factor, *N* normal, *n* number, *PLG* plasminogen

^a^The decrease in CFH serum concentration was mild (340 and 345 mg/L) and was not associated with potentially causative variants in CFH; ^b^Abnormal value was arbitrarily defined as above the upper limit of normal range; ^c^CFH serum concentration was normal

#### Genetic abnormalities

As in the cohort of patients with HUS, and as shown in Supplementary Table S5, many variants were also identified in the cohort of patients with C3G. Based on various parameters and their characteristics, they were considered benign or likely benign in 30% of patients and of unknown significance, risk-associated, likely pathogenic or pathogenic and in 80%. Among the latter group, and as depicted through Table 5, 30% of patients were also found to carry at least two variants in different alleles.

**Fig. 5:**
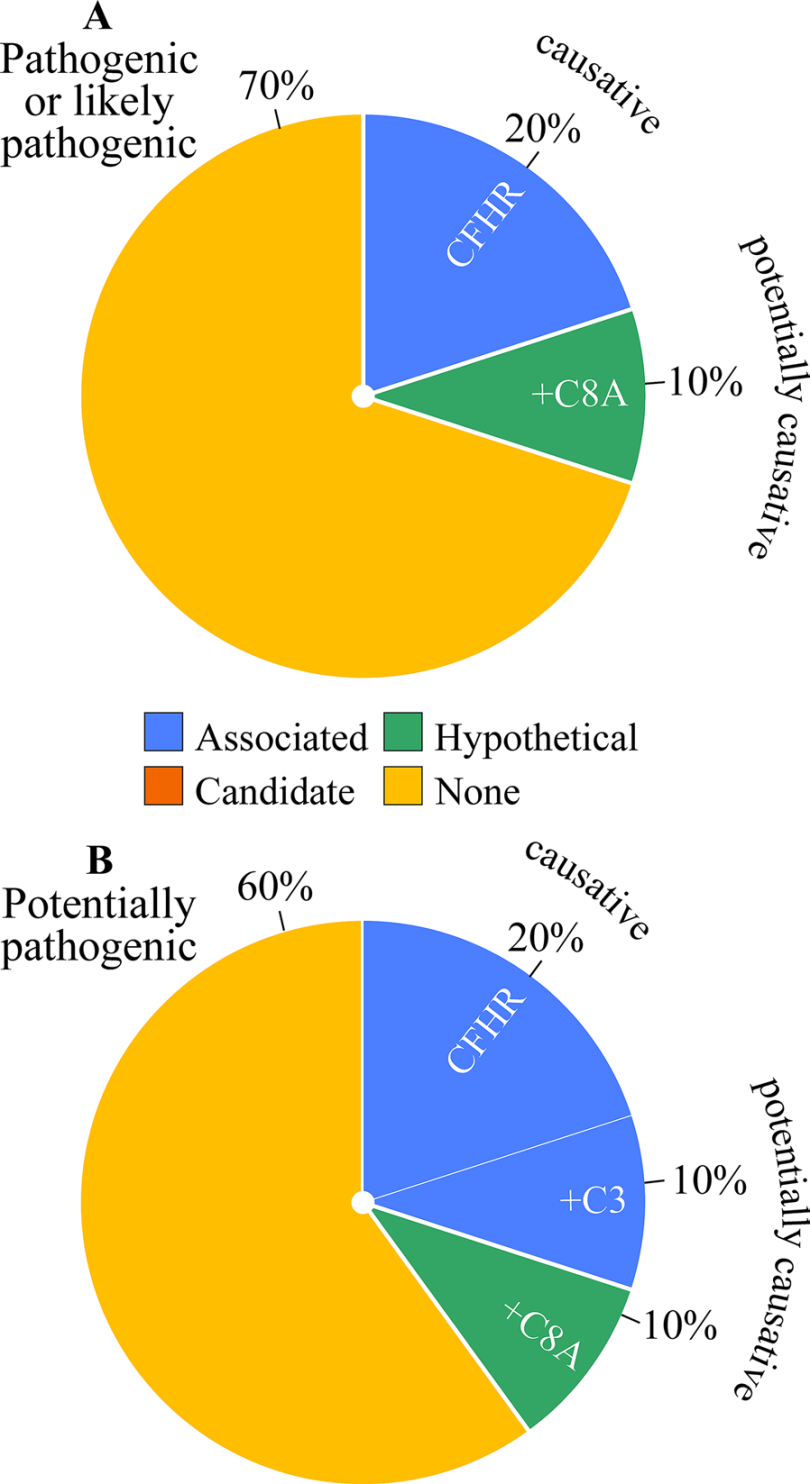
Distribution analysis of genetic variants identified in cohort with C3G. (A) Frequency of pathogenic, likely pathogenic or risk variants in genes tested. (B) Similar to (A) except for adding potentially pathogenic variants

The relative frequencies of the causative or potentially causative variants identified are illustrated through Fig. 5. As seen, a risk allele (CFHR3-1) is present in 20% of patients and a potentially causative variant (C8A) in 10%. This percentage increases to 40% when a potentially pathogenic variant in a causative gene (C3) is included. The nature and characteristics of the variants uncovered are presented more specifically in Table 8.

**Table 8:**
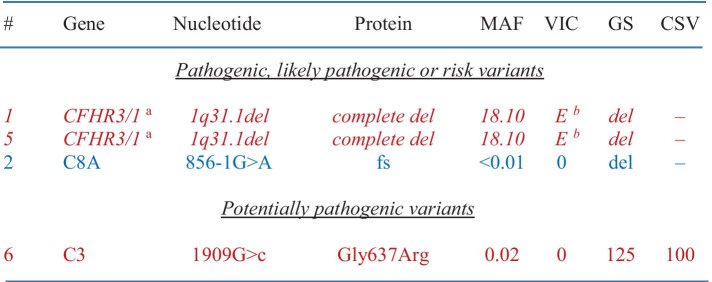
Variants of interest in cohort with C3G. One of the variants identified (shown in italic characters) is known to be associated with diseases of the alternative complement system and two of the variants identified (shown in non-italic characters) were considered potentially causative (shown in red characters for an associated gene and blue characters for a candidate or hypothetical gene). In the Table, parameters used to analyze the significance of each variant are also provided in the last four columns. Note that the variant in C8A is predicted to abolish the canonical 3' acceptor splice site (of intron 6) based on multiple splice site finders

#code assigned to case; Font and color code: in italic, known to be associated with C3G; not in italic, potentially causative; red, C3G-associated genes; blue, candidate or hypothetical genes for C3G

*CSV* conservation (% of mammalian species among > 50 different ones in which native residue in conserved), *del* deletion, *E* established risk or disease-causing variant, *fs* frameshift, *GS* Grantham score, *MAF* minor allele frequency, *VIC* variant in cases (as previously reported)

^a^Both alleles were affected by the same variant; ^b^Increased risk for complement-related disorders

### Additional studies

Going back to Tables 2 and 6, one can also compare the clinical features of the two cohorts. In patients with HUS, for instance, a higher prevalence of females, severe renal failure, and treatment with an anti-C5 antibody can be noted, and in patients with C3G, it is a higher prevalence of hypocomplementemia and severe proteinuria that can be noted.

In the HUS cohort, lastly, association analyses revealed that the risk of developing renal failure could not be predicted on the basis of age, sex, serum measurements (including LDH and C3 levels), and etiology (not shown). It could not be predicted either on the basis on age, sex, and serum measurements after stratification of the cohort by etiology.

## Discussion

In this study, we have described the clinical and genetic characteristics of two adult retrospective cohorts that were each affected by a disorder of the alternative complement pathway. One of these cohorts was unique by counting the largest number of individuals with HUS in a Canadian series and the largest number of individuals with biopsy-proven TMA in North America (*n* = 35). The other cohort was smaller in size as already stated.

Among the individuals who were affected by HUS, 11 were found to have primary aHUS. Yet, pathogenic variants in causative or risk-associated genes were identified in only one (9%). As for the other individuals, 24 were found to have non-primary HUS. For them, but perhaps less surprisingly and in keeping with more recent studies [39], pathogenic variants in the causative or risk-associated genes were also uncommon (17%). In our entire HUS cohort, the prevalence of known causative variants was thus quite low (14%) and etiology-independent.

Based on the International Registry of Recurrent and Familial HUS [1], the prevalence of causative variants in primary aHUS was found to be ~ 45% (out of 144 cases), i.e., over fivefold higher than in our cohort. In this Registry, however, it reached 75% in familial aHUS, which accounted for 30% of the cohort, and it was higher before the age of 18. In the light of such considerations and of our observations, the prevalence of causative variants in adult-onset sporadic primary aHUS is likely to be much lower than 45%.

Based on the same International Registry of Recurrent and Familial HUS, the prevalence of causative variants in secondary aHUS was found to be ~ 30% (out of 47 cases), i.e., still relatively high and twofold higher than in our cohort. Not impossibly, it would have been even lower after exclusion of children. It is yet noteworthy that the prevalence of causative variants in hypertension-associated aHUS was about the same in both the International Registry of Recurrent and Familial HUS and our cohort, i.e., ~ 13%.

As mentioned earlier, all cases of TMA in this series were proven by biopsy and unrelated to deficient ADAMTS13 activity. Even if the possibility of a misdiagnosis bias was thereby avoided, the prevalence of pathogenic variants in our population was still unexpectedly low. At the same time, one should keep in mind that our biopsy-based selection criterion could have also led to the inclusion of individuals with non-complement-mediated TMA and accounted for the outcome observed to some extent.

It is of notice that the North American population constituted only 14% of the International Registry of Recurrent and Familial HUS. If, in this context, it was also affected by a unique repertoire of causative variants, the prevalence of consequential genetic defects would be underestimated from lack of segregation or biological evidence. In fact, 20% of our patients with HUS were found to carry at least one potentially pathogenic variant in a causative or risk-associated gene and one of these variants was even proven to be disruptive through RT-PCR studies.

Remarkably, CD46 revealed normal in all of our patients with HUS even though it is the second most common causative gene in this disease [1, 2, 40]. Such a finding could suggest once again that the nature of the HUS-associated genes and of the variants at play differs as a function of the population tested, i.e., of age, gender, ethnicity, geographical origin, disease severity, and so on. At the same time, our cohort was not sufficiently powered to identify pathogenic or likely pathogenic variants in all of the known causative genes.

The candidate and hypothetical genes tested were not expected to harbor HUS-associated variants of known significance. However, they were sequenced to consolidate the status of certain genes or discover new ones. This goal was partially achieved by identifying pathogenic or potentially pathogenic variants in C3AR1, VWF, and C9 (among the candidates) and in C5, C7, and CR1 (among the new ones). Those in C9 and CR1 were of special interest given that they are likely to disrupt protein function.

In most of the previous cohort studies that were concerned with the genetics of HUS, the data shown were limited to pathogenic variants in the exome sequence of known causative or risk-associated genes [1, 2, 40]. For this reason, and as suggested by our findings, it is thus quite likely that many additional genes or variants of clinical significance would be identified through the use of extended panels. In this regard, recent studies have shown that wide exome scans in large cohorts of patients with kidney disorders could yield an unexpectedly high percentage of genetic diagnoses [41–44].

As for the in-depth biochemical or biological investigation of HUS in our cohort (Tables 3 and 7), it did not prove to be clinically useful. For instance, anti-CFH antibody titers were below threshold or only marginally increased in all individuals. Along the same line, serum CFH, CFB and CFI activity was not necessarily decreased because of pathogenic variants in the encoding gene. Lastly, serum or urinary C5b-9 was increased in most patients, but so were the serum LDH levels.

Two of our patients were diagnosed with malignant hypertension based on eye examination. However, the presence of grade III-IV retinopathy in these patients cannot incriminate hypertension as the sole cause of aHUS with certainty. In particular, the funduscopic presentation of non-hypertension-associated aHUS has still not been reported, abnormalities of the alternative complement pathway have been detected in many cases of so-called hypertension-associated aHUS [1, 15, 45, 46], severe hypertension rarely leads to aHUS, and renal failure in TMA-causing disorders is a major risk factor for the development of hypertension [47, 48].

As for the series of C3G, the CFHR3-1 locus was found to be affected by homozygous deletions in two patients, C8A by a pathogenic variant in one, and C3 by a potentially pathogenic variant in one. C8A was considered an interesting candidate gene given that C9 has been linked to aHUS in a few individuals and that both proteins are members of the membrane attack complex. Otherwise, anti-CFH antibodies titers were clearly increased (by more than 2 folds) in only two patients.

While variants in genes such as C3 or C9 should theoretically increase protein activity to render the complement system overly amplifiable and predispose to aHUS or C3G, they were found or predicted in this study to produce a large monoallelic deletion, i.e., a partial reduction in overall protein function. Yet, inherited haploinsufficiency in C3 has also been linked to aHUS based on one study [30], suggesting that it could have led to long-term adaptation mechanisms through which C5b-9 deposition is less tightly regulated. The molecular genetics of complement dysregulation are still poorly understood.

In future undertakings, our study will have to be expanded to determine whether many of the variants identified do affect protein function. However, such undertakings will probably prove to be quite challenging. In particular, variant-harboring proteins of the alternative and terminal complement pathway will have to be tested through in vitro studies to determine whether they actually exhibit or lead to gain-of-function activity. In addition, the general low penetrance of genetic defects in aHUS and C3G is often an obstacle to informative segregation studies [49].

On another note, it should be noticed that certain genes are difficult to sequence given that they come as several copies (those of the CFHR locus for instance) or share high homology with multiple pseudogenes. These factors may limit the diagnostic yield of genetic testing by next generation sequencing (NGS) as current technology is based on alignments of short sequences. Long-read NGS will probably improve our ability to detect causative variants in disorders of the alternative complement pathway and in many other disorders as well.

In conclusion, we have found that the prevalence of known causative variants in our adult population of HUS or C3G was less than 20%. However, we have also identified novel variants of potential significance and candidate genes that code for components of the terminal complement pathway. While an important challenge in complementopathies is to develop tailor-made interventions, it will probably not be met until these disorders are reclassified on the basis of molecular categories through the type of approaches that were exploited for the current study.

## Supplementary Information

Below is the link to the electronic supplementary material.

Supplementary file1 (DOCX 85.4 KB)

## Data Availability

Data will be made fully available upon request or through figshare if deemed warranted.

## Footnotes

1 https://www.proteinatlas.org/ENSG00000125730-C3/tissue
