# Supplementary material for: Genetic abnormalities in biopsy-proven, adult-onset hemolytic uremic syndrome and C3 glomerulopathy
Source: J Mol Med (Berl). 2021 Oct 29;100(2):269–84. doi: 10.1007/s00109-021-02102-1 (PMC8770394; doi:10.1007/s00109-021-02102-1)
Supplement: Supplementary file 1 — Supplementary file1 (DOCX 85.4 KB) [file 109_2021_2102_MOESM1_ESM.docx]

**Genetic abnormalities in biopsy-proven, adult-onset hemolytic uremic syndrome and C3 glomerulopathy**

^1^Ludwig Haydock, ^1,2^Alexandre P. Garneau, ^1^Laurence Tremblay, ^3^Hai-Yun Yen, ^3^Hanlin Gao, Raphaël Harrisson and ^1^Paul Isenring

^1^Nephrology Research Group, L’Hôtel-Dieu de Québec Research Center, Department of Medicine, Faculty of Medicine, Laval University, Québec (QC), Canada G1R2J6

^2^Cardiometabolic Axis, School of Kinesiology and Physical Activity Sciences, Faculty of Medicine, University of Montréal, 900, rue Saint-Denis, Montréal (QC), Canada H2X 0A9

^3^Fulgent Genetics, Temple City, CA, US 91780

**Running title:** Genetic abnormalities in complementopathies

**Address correspondence to:** Paul Isenring, MD, FRCPC, PhD

CHUQ-L’Hôtel-Dieu de Québec Research Center

10 Rue McMahon (Room 3852)

Québec (QC), Canada G1R2J6

Tel.: (418) 691-5151 (15477)

FAX: (418) 692-5795

E-mail: [paul.isenring@crhdq.ulaval.ca](mailto:paul.isenring@yale.edu)

**LEGENDS TO SUPPLEMENTARY TABLES**

Table S1. **Types of HUS and associated conditions.** The list of disorders presented is semi-exhaustive. Abbreviations: HUS, hemolytic uremic syndrome (HUS); HELLP, hemolysis, elevated liver enzymes and low platelet count.

Table S2. **Etiological investigation of patients with HUS.** All patients with diarrhea on admission had their stools examined for the presence of shigatoxins STX1 and STX2. Patients who were not tested for both serum anti-nuclear and anti-ENA antibody titers had no clinical evidence or histological signs (on their renal biopsies) of autoimmune disorders.

Table S3. **Variants identified in the cohort of patients with HUS.** Variants shown were considered of unknown significance, risk-associated, likely pathogenic or pathogenic. Unless mentioned otherwise, they were found to affect only one allele. As for the intronic variants listed, they are predicted to alter pre-mRNA splicing by creating a cryptic splice site (case #10) or abolishing an existing one (cases #10 and #26). All of the individuals who share the same variants among those reported previously and within our cohort (cases #19 and #21) are unrelated. Through in vitro studies, variant in case #5 was also found to alter protein expression and variant in case #12 to impair O-glycosylation. Note that the word “none” is used to indicate that the variants identified were benign or likely benign.

Table S4. **Etiological investigation of patients with C3G.** None of the patients had clinical evidence or histological signs (on their renal biopsies) of autoimmune disorders.

Table S5. **Variants identified in the cohort of patients with C3G.** Variants shown were considered of unknown significance, risk-associated, likely pathogenic or pathogenic. The variant in C8A is predicted to abolish the canonical 3’ acceptor splice site of intron 6. None of the VUS appear to have been assayed assayed through in vitro studies. Note that the word “none” is used once again to indicate that the variants identified were benign or likely benign.

Table S1. **Types of HUS and associated conditions.**

aHUS Etiology

STEC Shigatoxin

Primary Idiopathic

Secondary Autoimmune disorders

- Progressive systemic sclerosis

- Systemic lupus erythematosus

- Anti-phospholipid syndrome

- Primary glomerulonephritis

Drugs

- Mitomycin, gemcitabine

- Tyrosine kinase inhibitor

- Calcineurin inhibitors

- Interferon

- Quinine

- Interferon

- Oral contraceptive

Cancer

- Bone marrow transplant

- Solid cancers

Infections

- Cytomegalovirus

- Human immunodeficiency virus

- Influenza virus type A

- BK virus

- Hepatitis C virus

- Streptococcal pneumonia

Severe hypertension

Pregnancy

- Pre-eclampsia / eclampsia

- HELLP

Abbreviations: HUS, hemolytic uremic syndrome (HUS); HELLP, hemolysis, elevated liver enzymes and low platelet count.

Table S2. **Etiological investigation of patients with HUS.**

# HBV HCV HIV CMV ANA DNA ENA STX AD13 APL

1 − − NEG − BT BT BT − N BT

2 NEG NEG − − − − BT − − ^†^ −

3 NEG NEG − − BT BT − − N −

4 − − − − BT − BT ND − ^†^ −

5 NEG NEG − NEG BT BT BT ND N BT

6 NEG NEG NEG − BT BT BT − N BT

7 NEG NEG NEG − BT BT − − N −

8 NEG − NEG − BT − BT ND N BT

9 NEG NEG − NEG BT BT BT − N BT

10 NEG NEG NEG NEG − − − ND N −

11 − − − − BT − BT − N −

12 NEG NEG NEG − BT BT − ND N BT

13 NEG NEG NEG NEG BT − BT − N −

14 NEG NEG NEG − BT − BT − N −

15 NEG NEG NEG − AT ^8^ BT BT − N BT

16 NEG NEG − NEG BT − − − N BT

17 NEG − − NEG − − BT − N −

18 NEG NEG − − BT AT ^3^ BT − N AT ^10^

19 NEG NEG NEG NEG BT − BT − N BT

20 NEG NEG NEG − BT BT − ND N −

21 NEG NEG NEG NEG BT BT BT − N BT

22 NEG NEG NEG NEG BT − − − N −

23 NEG NEG NEG − BT BT BT − N BT

24 NEG NEG NEG − BT BT − − N BT

25 NEG NEG NEG − − − − ND N −

26 NEG NEG NEG − − − − ND N BT

27 − − − − BT BT − − N BT

28 NEG NEG NEG − BT − − − N −

29 NEG NEG NEG − BT − − ND N BT

30 NEG NEG NEG NEG BT BT BT − N −

31 NEG NEG NEG − BT − − D N BT

32 NEG NEG NEG NEG − − − − N −

33 NEG NEG NEG − BT − − − N −

34 NEG NEG − − BT BT BT ND N BT

35 NEG NEG NEG − − − − − N −

Signs: #, code assigned to case; ^†^, experienced recurrent aHUS; −, data unavailable. Abbreviations: AD13, ADAMTS13; ANA, antinuclear antibodies; APL, antiphospholipid antibodies (anti-cardiolipin and/or anti-β2GP1 IgG titers); AT, above titer (following number in superscript refers to fold increase relative to reference value); D, detected; DNA, anti-DNA titers; ENA, anti-ENA titers; HBV, hepatitis B virus; HCV, hepatitis C virus; HIV, human immunodeficiency virus; N, normal (were >59% in all cases); ND, not detected; NEG, negative (absence of acute or chronic infection by serology or PCR); STX, shigatoxin in stools.

Table S3. **Variants of significance or potential significance identified in the cohort of patients with HUS.** Categorizations are based on ACMG guidelines prior to reinterpretation.

# Gene nucleotide protein MAF VIC GS CSV ACMG

1 CFB 724A>C Ile242Leu 0.30 2 5 95 VUS

2 CR1 855_856insGC Trp286Alafs*71 0.01 0 del – VUS

3 None × × × × × × ×

4 CFH 3572C>T Ser119Leu 0.00 E 145 80 Patho

5 F12 –57G>C fs 1.83 0 del ^§^ del VUS

6 C7 2384C>T Ser795Leu 0.02 0 145 85 VUS

7 None × × × × × × ×

8 VWF 2771C>G Arg924Gln 1.89 0 43 94 VUS

ST3GAL1 262G>A Ala88Thr 0.12 0 58 73 VUS

9 CFHR3 839_840del Ile280Lysfs*7 0.18 1 del – VUS

CFHR3/1 1q31.1del Complete del 18.10 E del – Risk

10 C3 ^†^ 3810+205T>A Thr1272Serfs*39 0.00 0 del – VUS

C3 ^†^ 4855A>C Ser1619Arg 0.19 0 110 80 VUS

11 None × × × × × × ×

12 VWF c4457C>T Ser1486Leu 0.86 0 145 ^§^ 67 VUS

13 INF2 1640G>A Gly547Asp 0.08 0 94 94 VUS

VWF 3161C>T Thr1054Met 0.01 0 81 85 VUS

14 DGKE 1513C>A His505Asn <0.01 0 68 100 VUS

15 None × × × × × × ×

16 None × × × × × × ×

17 None × × × × × × ×

18 CFB 1407C>G Ile469Met 0.02 0 10 64 VUS

19 CFH 3581G>A Gly1194Asp <0.01 2 94 65 VUS

20 PLG 782G>A Arg261His 0.48 0 29 99 VUS

C5 3199A>C Ser1067Arg 0.02 0 110 98 VUS

21 CFH ^‡^ 3581G>A Gly1194Asp <0.01 2 94 65 VUS

22 None × × × × × × ×

23 None × × × × × × ×

24 None × × × × × × ×

25 CFI 1657C>T Pro553Ser ^⸸^ 0.27 M 74 85 VUS

C9 1039_1042del Ser347Alafs*5 0.00 0 del – L-patho

26 CD55 691A>G Ile231Val 0.02 0 29 87 VUS

CFHR4 799+3A>C fs 0.25 0 del – VUS

27 None × × × × × × ×

28 CFI E9_E13del Large del / fs <0.01 E del – Risk

29 C3AR1 1020G>T Arg340Ser 0.01 0 110 – VUS

30 VWF 1625G>C Ala542Gly 0.14 0 60 39 VUS

C3AR1 561T>G Phe187Leu 0.01 0 22 – VUS

31 CFH 661A>G Ile221Val <0.01 E 29 49 Risk

32 None × × × × × × ×

33 None × × × × × × ×

34 None × × × × × × ×

35 CFH 2823_2825del Val942del <0.01 0 del – VUS

VWF 7135C>T Arg2379Cys <0.01 0 180 100 VUS

VWF 7988G>C Arg2663Pro 0.23 0 103 86 VUS

C5 3029C>T Ala1010Val 0.12 0 64 100 VUS

Signs: #, code assigned to case; ^†^, one was in cis and the other in trans, ^‡^, homozygote; ^§^, reported to disrupt protein function in vitro; ^⸸^, associated with aHUS with variants in CD46 and/or CFH; −, data unavailable; ×, variants were considered benign or likely benign. Abbreviations: ACMG, American College of Medical Genetics; CSV, conservation (% of mammalian species among >50 different ones in which native residue in conserved); del, deletion; E, established risk or disease-causing variant; fs, frame shift; GS, Grantham score; L-patho, likely pathogenic; M, multiple; MAF, minor allele frequency; patho, pathogenic; VIC, variant in cases (as previously reported); VUS, variant of unknown significance.

Table S4. **Etiological investigation of patients with C3G.**

# HBV HCV HIV CMV ANA DNA ENA ASLO MS APL

1 NEG NEG NEG − BT BT BT AT ND BT

2 NEG NEG NEG NEG BT BT BT AT ND BT

3 NEG NEG NEG NEG BT − BT − ND −

4 NEG NEG − − BT − − − ND −

5 NEG NEG NEG − BT BT BT − ND BT

6 NEG NEG NEG NEG − − − − D BT

7 NEG NEG POS NEG BT − − − ND −

8 NEG NEG NEG NEG BT BT − BT D −

9 NEG NEG NEG − BT BT BT BT D BT

10 NEG NEG − − BT − − − D −

Signs: #, code assigned to case; −, data unavailable. Abbreviations: ANA, antinuclear antibodies; APL, antiphospholipid antibodies (anti-cardiolipin or anti-β2GP1 IgG titers); ASLO, antistreptolysine O; AT, above titer; CMV, cytomegalovirus; D, detected; DNA, anti-DNA titers; ENA, anti-ENA titers; HBV, hepatitis B virus; HCV, hepatitis C virus; HIV, human immunodeficiency virus; MS, monoclonal spike; ND, not detected; NEG, negative (absence of acute or chronic infection by serology or PCR); STX, shigatoxin in stools.

Table S5. **Variants identified in the cohort of patients with C3G.** Categorizations are based on ACMG guidelines prior to reinterpretation.

# Gene nucleotide protein MAF VIC GS CSV ACMG

1 CFHR3/1 ^†^ 1q31.1del complete del 18.10 E del – Risk

2 C8A 856-1G>A fs <0.01 0 del – L-patho

3 C3 463A>C Lys155Gln 0.54 0 53 56 VUS

4 CFH 3226C>G Gln1076Glu 0.16 1 29 10 VUS

C3AR1 1400A>G His467Arg <0.01 0 29 – VUS

5 CFHR3/1 ^†^ 1q31.1del Complete del 18.10 E del – Risk

6 C3 1909G>C Gly637Arg 0.02 0 125 100 VUS

7 None × × × × × × ×

8 None × × × × × × ×

9 PLG 1997T>C Ile666Thr 0.02 0 89 97 VUS

C7 1852C>T Arg618Trp 0.14 0 101 23 VUS

10 None × × × × × × ×

Signs: #, code assigned to case; ^†^, homozygote; −, data unavailable; ×, variants were considered benign or likely benign. Abbreviations: ACMG, American College of Medical Genetics; CSV, conservation (% of mammalian species among >50 different ones in which native residue in conserved); del, deletion; E, established risk or disease-causing variant; fs, frame shift; GS, Grantham score; L-patho, likely pathogenic; MAF, minor allele frequency; patho, pathogenic; VIC, variant in cases (as previously reported); VUS, variant of unknown significance.
